# Pharmacological Modulation of Three Modalities of CA1 Hippocampal Long-Term Potentiation in the Ts65Dn Mouse Model of Down Syndrome

**DOI:** 10.1155/2018/9235796

**Published:** 2018-04-10

**Authors:** Jonah J. Scott-McKean, Adriano L. Roque, Krystyna Surewicz, Mark W. Johnson, Witold K. Surewicz, Alberto C. S. Costa

**Affiliations:** ^1^Division of Pediatric Neurology, Department of Pediatrics, Case Western Reserve University, Cleveland, OH 44106, USA; ^2^Postgraduate Program in Medicine, Cardiology, Federal University of Sao Paulo, 04024-002 Sao Paulo, SP, Brazil; ^3^Department of Physiology and Biophysics, Case Western Reserve University, Cleveland, OH 44106, USA; ^4^Department of Psychiatry, Case Western Reserve University, Cleveland, OH 44106, USA

## Abstract

The Ts65Dn mouse is the most studied animal model of Down syndrome. Past research has shown a significant reduction in CA1 hippocampal long-term potentiation (LTP) induced by theta-burst stimulation (TBS), but not in LTP induced by high-frequency stimulation (HFS), in slices from Ts65Dn mice compared with euploid mouse-derived slices. Additionally, therapeutically relevant doses of the drug memantine were shown to rescue learning and memory deficits in Ts65Dn mice. Here, we observed that 1 *μ*M memantine had no detectable effect on HFS-induced LTP in either Ts65Dn- or control-derived slices, but it rescued TBS-induced LTP in Ts65Dn-derived slices to control euploid levels. Then, we assessed LTP induced by four HFS (4xHFS) and found that this form of LTP was significantly depressed in Ts65Dn slices when compared with LTP in euploid control slices. Memantine, however, did not rescue this phenotype. Because 4xHFS-induced LTP had not yet been characterized in Ts65Dn mice, we also investigated the effects of picrotoxin, amyloid beta oligomers, and soluble recombinant human prion protein (rPrP) on this form of LTP. Whereas ≥10 *μ*M picrotoxin increased LTP to control levels, it also caused seizure-like oscillations. Neither amyloid beta oligomers nor rPrP had any effect on 4xHFS-induced LTP in Ts65Dn-derived slices.

## 1. Introduction

Down syndrome (DS) is the set of phenotypic features of variable expressivity that results from an extra copy of chromosome 21 (trisomy 21) and is the most common genetically defined cause of intellectual disability [1, 2]. Persons with DS typically display moderate intellectual disability [3], with disproportionate deficits in hippocampus-dependent and executive functions [4–6]. Individuals with DS exhibit a neuropathology indistinguishable from Alzheimer disease (AD) by age 40 [7] and high prevalence of early-onset dementia in their fifth and sixth decades of life [8].

The Ts65Dn mouse is the most widely researched mouse model for DS and the most complete in terms of displaying phenotypes mimicking what is observed in persons with DS (for reviews see [9, 10]). In attempts to understand the neurobiological basis for the cognitive deficits associated with DS, much emphasis has been placed on the study of synaptic plasticity in these animals, mostly long-term potentiation (LTP), and, to a smaller extent, long-term depression (LTD). This is because these forms of synaptic plasticity are thought to engage some of the major cellular and circuit-level mechanisms underlying learning and memory [11, 12].

Using brain slice techniques, Kleschevnikov et al. [13] were the first to demonstrate significant deficits in the induction of LTP in the dentate gyrus of Ts65Dn mice, whereas Costa and Grybko [14] were the first to show unequivocally LTP deficits in the CA1 region of the hippocampus. Siarey et al. [15, 16] were the first to demonstrate enhanced levels of CA1 LTD in Ts65Dn mice, and Scott-McKean and Costa [17] established that this phenomenon is associated with N-methyl-D-aspartic acid (NMDA) receptor dysfunction in these animals.

LTP can be induced by a large number of electrical and pharmacological stimulation protocols, which may be custom-tailored by an individual research group to answer very specific questions about synaptic plasticity mechanisms in a given system. For the purposes of surveying the general integrity of synaptic plasticity in hippocampal slices from mouse models of neurological and psychiatric disorders, however, three broad categories of induction protocols can capture the most significant features one typically associates with LTP. The first protocol involves a single high-frequency stimulation (HFS); generally, a train of stimuli applied at high frequency designed to produce a strong tetanic stimulation of a single synaptic pathway. In the Schaffer collateral pathway in the CA1 region of the hippocampus, such modality of LTP is NMDA receptor-dependent, entails the activation of CaMKII and PKC, is independent of protein synthesis, and lasts at most for a few hours [18, 19]. Although Siarey et al. [20] had initially observed a deficit in such form of CA1 LTP in Ts65Dn mice compared with euploid control mice, additional studies, beginning with Costa and Grybko [14], found that this form of LTP is unaffected in Ts65Dn mice.

The second commonly used LTP induction protocol involves multiple short bursts of 3-4 stimuli separated by a fixed interval designed to mimic the physiological situation in which presynaptic cells generate short bursts of synaptic activity driven by an underlying circuit activation pattern, typically a theta-frequency wave. The LTP resulting from such theta-burst stimulation (TBS) induction protocol has many of the characteristics of HFS. However, because of the 200-millisecond intervals between bursts of stimulation, it is thought to be more dependent on the activation of GABAergic interneurons than HFS [21]. Costa and Grybko [14] observed a small but significant reduction on the levels of CA1 LTP induced by TBS in hippocampal slices from Ts65Dn mice compared with euploid control mice.

The third CA1 LTP induction protocol involves the use of multiple (typically 3 or 4) HFS separated by an interval of a few minutes, which produces a robust and long-lasting (days, weeks, or months) enhancement in synaptic transmission. The resulting late-phase LTP (L-LTP) requires gene transcription and protein synthesis in the postsynaptic neuron [22]. Therefore, this form of LTP can be seen as an informative assay of the integrity of the essential transcriptional and translational machinery in neuronal cells.

In the present study, we probed the three modalities of CA1 LTP described above in hippocampal slices derived from Ts65Dn and euploid control mice. The reason to revisit the first two forms of LTP in these mice was to investigate whether therapeutically relevant levels of the uncompetitive NMDA receptor antagonist, memantine, could interfere with the properties of either of these NMDA receptor-dependent phenomena.

We found this investigation to be highly relevant to broaden our understanding of the role of NMDA receptor dysfunction in the pathogenesis of DS, particularly in light of work showing that memantine can rescue learning and memory deficits in Ts65Dn mice [23–25]. In addition, this drug was also shown to rescue the exaggerated levels of NMDA receptor-dependent CA1 LTD in Ts65Dn mice [17]. These preclinical data led to the design of a pilot clinical trial of memantine aiming at enhancing the cognitive abilities of individuals with DS [26] and a follow-up, Phase II clinical trial of memantine in adolescents and young adults with DS (“NCT02304302” at http://www.clinicaltrials.gov), which is underway.

LTP induced by multiple HFS in CA1 hippocampal slices (and the resulting L-LTP) had not yet been characterized in Ts65Dn mice. Therefore, in addition to testing the effects of memantine, we decided to expand our investigation to include three additional molecules of interest to the study of DS that could potentially shed some light on this specific form of LTP in Ts65Dn mice: (1) picrotoxin; (2) amyloid beta (A*β*) oligomers; and (3) soluble (membrane anchor-free) recombinant human prion protein (rPrP).

## 2. Materials and Methods

### 2.1. Animals

The original production of the segmental trisomy Ts65Dn has been well described in the literature [27]. Experimental mice were generated by repeated backcrossing of Ts65Dn females to C57BL/6JEiJ × C3Sn.BLiA-Pde6b+/D F1 hybrid males (as described in [28]) at the Case Western Reserve University's Animal Resource Center. Male euploid littermates of Ts65Dn mice were used as controls. Animals from the same litter and sex were housed in the same cage and maintained in a 12 : 12-hour light/dark schedule (lights on at 6:00 a.m.) with ad libitum access to food and water. Adult male Ts65Dn and euploid littermate controls age 6–9 months were used in the experiments described here. All experimental methods have received the approval from the Case Western Reserve University's Animal Care and Use Committee.

### 2.2. Slice Preparation

Mice were decapitated under halothane anesthesia and their brains were rapidly harvested for dissection in ice-cold artificial cerebral spinal fluid (aCSF) (in mM: 120 NaCl, 3.5 KCl, 2.5 CaCl_2_, 1.3 MgSO_4_, 1.25 NaH_2_PO_4_, 26 NaHCO_3_, and 10 D-glucose, saturated with 95% O_2_ and 5% CO_2_). Slices were then cut with a vibrating blade microtome (VT 1000s, Leica, Bannockburn, IL) into transverse, 400 *μ*m thick slices. Hippocampi were dissected out and moved into a holding chamber containing oxygenated aCSF and allowed to recover for at least an hour at room temperature. For experiments with picrotoxin and A*β* oligomers, hippocampal slices were placed in the recording chambers soon after this recovery period. For experiments with memantine, half of the slices stayed in the original holding chamber and half were moved to a second holding chamber with oxygenated aCSF containing 1, 3, or 10 *μ*M memantine. In these experiments, both sets of hippocampal slices were maintained in their respective holding chambers for an additional 4 hours before being placed in the recording chamber.

### 2.3. Drug and Experimental Compounds

Stock solutions of memantine (Sigma, St. Louis, MO) were dissolved in standard aCSF (see above) at 1, 3, or 10 mM, stored at −80°C until the day of experiments, and diluted 1 : 1000 to a final concentration of 1, 3, or 10 *μ*M on the day of experiments. For the two lowest concentrations of picrotoxin (Sigma) used here (0.1 and 1 *μ*M), 10x stock solutions were prepared (1 and 10 *μ*M, resp.) and diluted to the appropriate concentration on the day of the experiment. Given that picrotoxin does not dissolve in water at high concentrations, 10 and 100 *μ*M were dissolved in aCSF on the day of the experiment. Bacterial expression and purification of rPrP were performed as described previously [29], and the protein concentration was determined by measuring absorbance at 276 nm using the appropriate extinction coefficients.

### 2.4. Preparation of A*β* Oligomers and rPrP

Preparation of A*β*1-42 oligomers (referred to, throughout the text, as A*β* oligomers) and rPrP has been previously described [30]. Briefly, human A*β*1-42 (rPeptide, LLC, Bogart, GA) was solubilized in hexafluoro-2-propanol, divided into aliquots, and after solvent evaporation, was stored at −80°C. Immediately before use, the peptide was dissolved in 10 mM NaOH, into a concentration of 400 *μ*M, and subjected to 10 cycles of 10-second sonication on ice to remove any residual aggregates. Disaggregated A*β* was diluted in 10 mM sodium phosphate, pH 7.4, to a concentration of 100 *μ*M. To prepare soluble A*β* oligomers, the peptide (20 *μ*M) was incubated in 50 mM sodium phosphate buffer, pH 7.4, for 3 hours at 37°C. Once every 20 minutes, the plates were subjected to shaking for 10 seconds. As shown previously, these experimental conditions result in A*β* preparations that are highly enriched in soluble oligomers [30]. Finally, rPrP was added to freshly disaggregated A*β* and these mixtures were incubated as described above for A*β* alone.

### 2.5. Electrophysiology

Parallel recordings of treated (memantine, picrotoxin, A*β* oligomers, rPrP, or A*β* oligomers + rPrP) and untreated hippocampal slices from the same animal were performed using a customized electrophysiology setup. In all experiments, one hippocampal slice was used per animal per condition. Field excitatory postsynaptic potentials (fEPSPs) were recorded with Ag/AgCl recording electrodes through thin-walled, 1.5 mm, WPI borosilicate glass micropipettes filled with aCSF (3–5 MΩ resistance), inserted into the CA1 region of the hippocampus. Two fine bipolar platinum/iridium electrodes (FHC, Bowdoinham, ME) were positioned on the CA1 Schaffer collateral fibers at opposite sides of the recording pipette. Stimulation intensity was adjusted to 40–50% of the amplitude that is required to produce population spikes. For experiments probing the effects of memantine or picrotoxin, a stable baseline of synaptic transmission was established for 20 minutes prior to the induction of LTP or L-LTP. LTP was induced by high-frequency stimulation (HFS; 100 Hz for 1 s), theta-burst stimulation (TBS; 5 trains of 4 pulses at 100 Hz, 200 ms intertrain interval), or four stimulus trains (4xHFS; with a 5-minute intertrain interval) in the CA1 region of the hippocampus in Ts65Dn- and euploid control-derived hippocampal slices. In some 4xHFS LTP experiments, we quantified the mean levels of early-phase LTP (E-LTP) at 60 minutes and/or late-phase LTP (L-LTP) at 180-minute poststimulation. To assess the potential modulatory effects of A*β* oligomer on 4xHFS-induced LTP in Ts65Dn-derived and euploid control-derived hippocampal slices, a stable baseline of synaptic transmission was established for 10 minutes before the bath application of A*β* oligomers (500 nM) alone or A*β* (500 nM) mixed with rPrP (100 nM). While slices were superfused with these test molecules, a second stable synaptic transmission baseline was established for 10 minutes prior to induction of LTP by 4xHFS. Bath perfusion of the test molecules continued for 10 minutes after LTP induction. To reduce the use of A*β* oligomers, aCSF was recirculated during this time.

Signals from the recording electrode were amplified 1000 times (Brownlee Precision Electrophysiology Amplifier Model 440, San Jose, CA), low-pass filtered (8-pole Bessel) at 2 kHz, and digitized at 20 kHz by a Digidata digitizer (1322A, Axon Instruments) into a Microsoft Windows-based computer. PCLAMP software (PCLAMP 8.2, Axon Instruments) was used for data acquisition and offline data analysis.

### 2.6. Statistics

Synaptic efficacy was determined by the slope of fEPSPs normalized to the mean value of fEPSP slopes recorded prior to the induction of LTP or L-LTP. Comparisons between mean levels of LTP in slices from the two genotypes investigated (Ts65Dn and euploid control), subjected to 1 *μ*M memantine, were done by two-way analysis of variance (ANOVA) (Statistica, version 12, Dell Inc., Tulsa, OK). Simple comparisons between the mean levels of 4xHFS-induced LTP at 60-minute and 180-minute poststimulation in slices from the two genotypes were assessed using two-tailed unpaired *t*-tests with Welch's correction (GraphPad Prism 7.0, GraphPad Software Inc., San Diego, CA, USA). Comparisons of mean levels of L-LTP in slices in a given genotype for three different pharmacological treatments (memantine, picrotoxin, and a*β* oligomers + rPrP) were done by one-way ANOVA (Statistica). Comparisons of mean normalized levels of L-LTP between the two genotypes, when subjected to progressively higher levels of memantine or picrotoxin, were performed by repeated measures ANOVA (RM-ANOVA) (Statistica). Comparisons of mean normalized levels of 4xHFS-induced E-LTP between the two genotypes, when subjected to a*β* oligomers, rPrP, or a*β* oligomers + rPrP, were performed by two-way ANOVA (Statistica). When ANOVA detected either a main factorial effect or an interaction between factors, it was followed by post hoc multiple comparisons using the Fisher's least significant difference (LSD) (Statistica). For all comparisons, a “*p*” value of <0.05 was selected as the criterion for statistical significance.

## 3. Results

### 3.1. The Effects of Memantine on HFS- and TBS-Induced LTP

**Figure 1:**
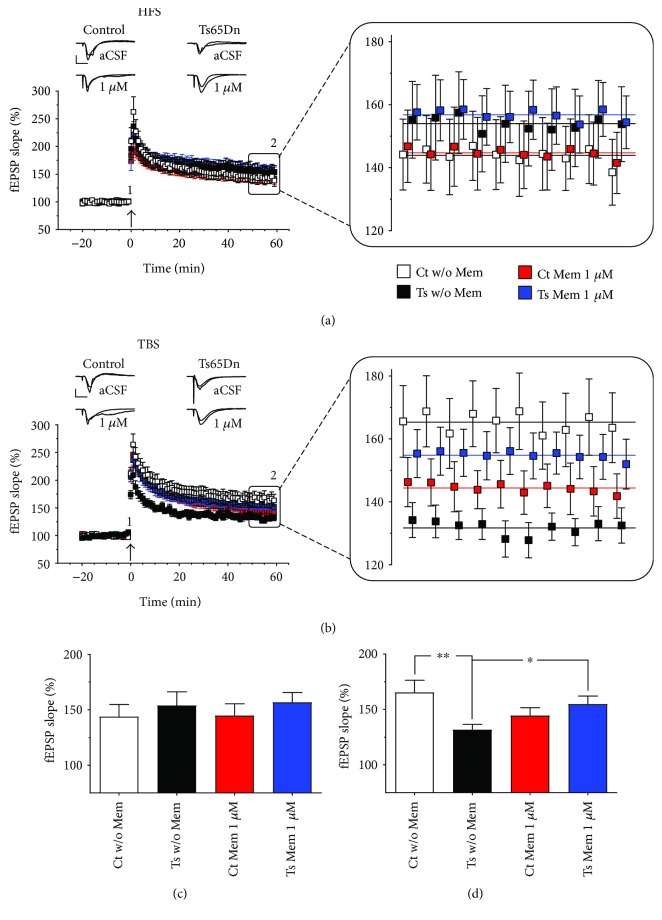
The effects of 1 *μ*M memantine on HFS- and TBS-induced LTP in hippocampal slices from Ts65Dn and euploid control mice. (a) Normalized fEPSP slopes of HFS-induced LTP and (b) TBS-induced LTP. Magnification of the last 10 minutes of recording. Colored lines represent the calculated mean fEPSP slopes for the last 10 minutes of recording. (c, d) Summary graph representation of the LTP data (mean fEPSP slope during the last 10 min of recording for each hippocampal slice). When used at the therapeutically relevant concentration of 1 *μ*M, memantine did not alter the induction or maintenance of HFS-induced LTP but it rescued TBS-induced LTP to control euploid levels in slices from Ts65Dn mice. *p* < 0.05 and *p* < 0.01 are represented by ^∗^ and ^∗∗^, respectively. Number of slices (animals) for HFS (Ct w/o Mem (*n* = 11), Ct Mem (*n* = 11), Ts w/o Mem (*n* = 11), and Ts Mem (*n* = 11)) and TBS (Ct w/o Mem (*n* = 13), Ct Mem (*n* = 13), Ts w/o Mem (*n* = 13), and Ts Mem (*n* = 15)). Error bars represent SEM. Arrow indicates LTP induction, representative traces show synaptic response during baseline (1) and at end of recording (2). Scale bars represent 1 mV (horizontal) and 10 ms (vertical).

In this subsection, we describe the results of experiments in which we compared levels of HFS-induced and TBS-induced CA1 LTP in Ts65Dn- and euploid control-derived untreated slices versus slices treated with 1 *μ*M memantine. For HFS-induced LTP, in agreement with the previous work [14], we found no significant difference between levels of LTP in slices from Ts65Dn mice compared with those from euploid littermate control mice (Figures 1(a) and 1(c)). In addition, we found that treatment with 1 *μ*M memantine did not significantly alter the induction or maintenance of this form of LTP in either Ts65Dn or euploid littermate control-derived slices (Figures 1(a) and 1(c)). Two-way ANOVA failed to detect any significant genotype (*F*_(1,40)_ = 1.058, *p* = 0.31) or treatment (*F*_(1,40)_ = 0.030, *p* = 0.86) effect, or any interaction between genotype and treatment (*F*_(1,40)_ = 0.0084, *p* = 0.93).

For TBS-induced LTP, our results were also in agreement with the previous work, with Ts65Dn-derived slices displaying decreased LTP levels in relation to those from euploid control mice (Figures 1(b) and 1(d)). In addition, we found that 1 *μ*M memantine increased the mean level of TBS-induced LTP in Ts65Dn-derived slices to that recorded in euploid control slices. Although, two-way ANOVA detected no significant genotype (*F*_(1,50)_ = 2.12, *p* = 0.15) or treatment (*F*_(1,50)_ = 0.019, *p* = 0.89) effect, there was a significant genotype × treatment interaction (*F*_(1,50)_ = 7.69, *p* = 0.0078). Accordingly, post hoc analysis indicated that untreated Ts65Dn-derived slices had a decreased mean level of LTP when compared with untreated euploid control-derived slices (*p* = 0.0049) and with memantine-treated Ts65Dn-derived slices (*p* = 0.041). Additionally, post hoc analysis showed that the mean level of LTP in memantine-treated Ts65Dn-derived slices was not significantly lower than untreated euploid control-derived slices (*p* = 0.35).

### 3.2. The Effects of Memantine on L-LTP

**Figure 2:**
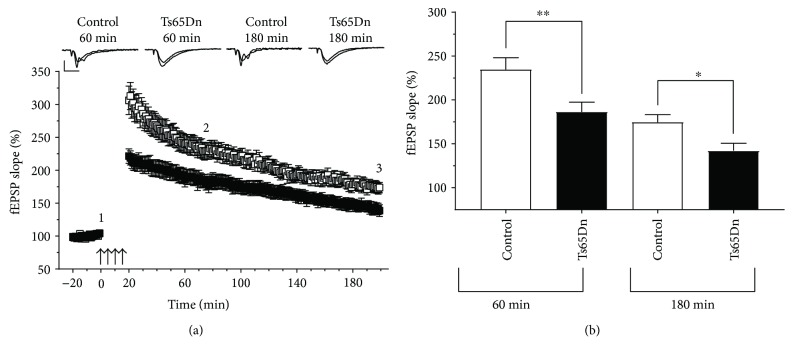
Ts65Dn hippocampal slices show reduced 4xHFS-induced LTP compared with slices from euploid control mice. (a) Comparisons of 4xHFS-induced L-LTP on euploid control-derived slices versus Ts65Dn-derived slices at 60 min and 180 min after induction. (b) Summary graph shows a reduction of E-LTP in Ts65Dn slices at 60 min and a reduction at 180 min after induction of L-LTP. *p* < 0.05 and *p* < 0.01 are represented by ^∗^ and ^∗∗^, respectively. Number of slices (animals) for control (*n* = 16) and Ts65Dn (*n* = 15). Error bars represent SEM. Arrows indicates LTP induction (4xHFS; with a 5 min intertrain intervals), representative traces show synaptic response during baseline (1) and at end of recording (2). Scale bars represent 1 mV (horizontal) and 10 ms (vertical).

Here, we describe the effects of memantine on L-LTP induced by 4xHFS. Because this specific form of LTP had not yet been characterized in Ts65Dn mice, we first compared the mean level of LTP in Ts65Dn-derived hippocampal slices with that in euploid control slices. In these experiments, we observed reduced levels of L-LTP in slices from Ts65Dn mice when compared with those of euploid control mice at both 60 min and 180 min after LTP induction (Figure 2(a)). Unpaired Student *t*-test comparisons of the mean levels of LTP showed a significant difference between control- (234.70 ± 13.43; *n* = 16) and Ts65Dn-derived hippocampal slices (186.30 ± 11.20; *n* = 15) (*t* = 2.77; df = 28.4; *p* = 0.0097) at 60 min after LTP induction (Figure 2(b)). Such genotype-dependent significant difference in mean levels of LTP persisted even at 180 min after LTP induction (Figure 2(b)) (174.70 ± 8.50; *N* = 16; and 142.20 ± 8.50; *N* = 15; for euploid control- and Ts65Dn-derived slices, resp.) (*t* = 2.70; df = 28.97; *p* = 0.011).

**Figure 3:**
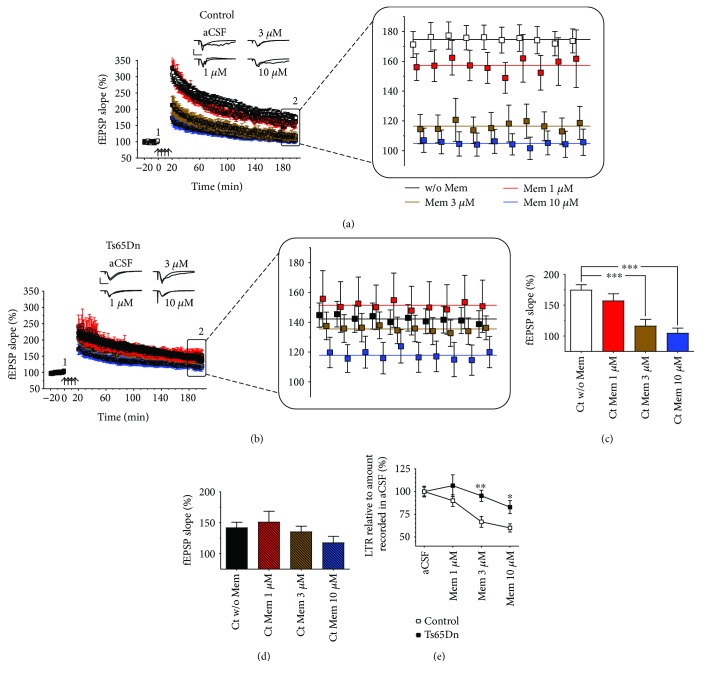
The effects of memantine on L-LTP. (a) Comparison of the effects of memantine (1, 3, and 10 *μ*M) on 4xHFS-induced L-LTP in euploid control-derived slices and (b) Ts65Dn-derived slices. Magnification of the final 10 minutes of recording. Colored lines represent the mean fEPSP slopes calculated for the last 10 minutes of recording. (c) Summary graph of euploid littermate control L-LTP data and (d) Ts65Dn L-LTP data (mean fEPSP slope during the last 10 min for each recording condition). Memantine at 3 and 10 *μ*M blocked the induction of L-LTP in euploid control slices and had no effect on Ts65Dn-derived slices. (e) Normalized data shows a greater inhibited L-LTP in euploid control slices at 3 and 10 *μ*M when compared to Ts65Dn slices. *p* < 0.05, *p* < 0.01, and *p* < 0.001 are represented by ^∗^, ^∗∗^, ^∗∗∗^, respectively. Number of slices (animals) for control (without mem (*n* = 16), mem 1 *μ*m (*n* = 13), mem 3 *μ*m (*n* = 10), and mem 10 *μ*m (*n* = 10)) and Ts65Dn (without mem (*n* = 15), mem 1 *μ*m (*n* = 11), mem 3 *μ*m *n* = 10, and mem 10 *μ*m (*n* = 10)). Error bars represent SEM. Arrows indicates LTP induction (4xHFS; with a 5 min intertrain intervals), representative traces show synaptic response during baseline (1) and at end of recording (2). Scale bars represent 1 mV (horizontal) and 10 ms (vertical).

We then investigated the potential effects of memantine treatment on 4xHFS-induced L-LTP in Ts65Dn and euploid control slices. We used three concentrations of memantine (1, 3, and 10 *μ*M) to assess the concentration dependence of any potential effect of this drug (Figures 3(a) and 3(b)). Similar to the HFS-induced LTP and TBS-induced CA1 LTP, both Ts65Dn- and euploid control-derived hippocampal slices were pretreated with the appropriate concentration of memantine 4 h before the start of the recording session. RM-ANOVA indicated that memantine (which was also continuously bath-perfused during the recording sessions) significantly inhibited the induction of L-LTP in euploid control-derived slices (*F*_(3,45)_ = 11.55, *p* = 0.00001) (Figure 3(c)), but did not significantly affect the induction of L-LTP in Ts65Dn-derived slices (*F*_(3,42)_ = 1.40, *p* = 0.26) (Figure 3(d)). Post hoc analysis indicates that memantine significantly decreased the level of L-LTP induced in euploid control-derived slices at the concentrations of 3 *μ*M (*p* = 0.00011) and 10 *μ*M (*p* = 0.000007) (Figure 3(c)). Therefore, in contrast to TBS-induced LTP, 1 *μ*M memantine did not increase the mean level of L-LTP seen in Ts65Dn slices, however, this drug still had a genotype-dependent effect by not reducing the levels of L-LTP in Ts65Dn slices (Figure 3(d)).

As an alternative means of visualizing the genotype-dependent effects of memantine on L-LTP mentioned in the previous paragraph, the data was normalized to the untreated genotype-specific levels. (In other words, the mean L-LTP levels in both euploid control- and Ts65Dn-derived untreated slices were set as 100% and the mean L-LTP levels for memantine-treated slices were obtained by dividing the LTP level for each concentration of memantine by its genotype-specific untreated levels and multiplying the result by 100.) As suspected, RM-ANOVA of the resulting data unveiled a significant genotype (*F*_(1,18)_ = 9.58, *p* = 0.0062) and dose (*F*_(3,54)_ = 6.12, *p* = 0.0012) dependence. However, no significant dose × genotype interaction was found (*F*_(3,54)_ = 2.41, *p* = 0.078). Here again, post hoc analysis showed that memantine at the doses 3 *μ*M (*p* = 0.0091) and 10 *μ*M (*p* = 0.036) reduced the level of L-LTP induced in euploid control-derived slices, but not in Ts65Dn-derived slices.

### 3.3. High Doses of Picrotoxin Increase the Levels of L-LTP in Ts65Dn-Derived Slices

Kleschevnikov et al. [13] have shown that reduced levels of HFS-induced LTP in the dentate gyrus of Ts65Dn mice can be pharmacologically enhanced by the antagonism of gamma-aminobutyric acid receptor type A (GABA_A_)-mediated synaptic inhibition with picrotoxin. A similar effect of picrotoxin has also been shown on CA1, TBS-induced LTP in Ts65Dn-derived hippocampal slices [14].

**Figure 4:**
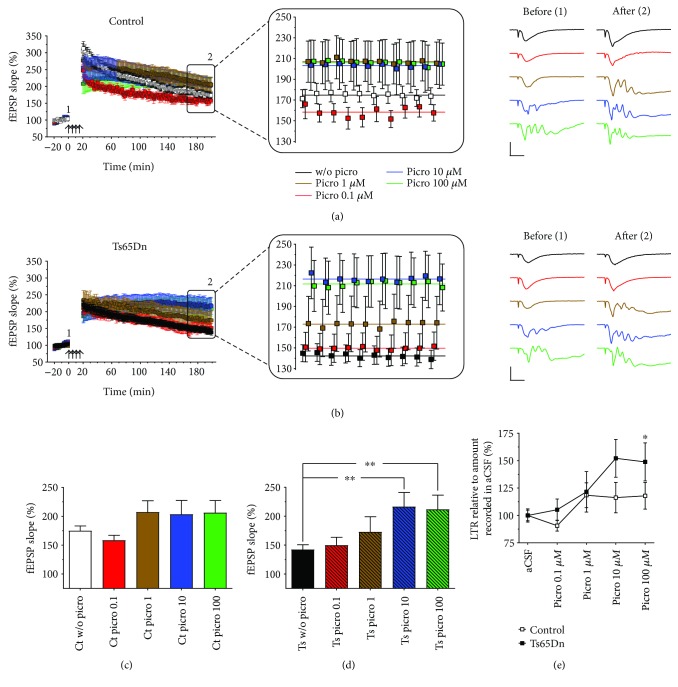
Dose dependence of the effects of picrotoxin on L-LTP induced by 4xHFS. (a) Comparisons of the effects of picrotoxin (0.1, 1, 10, and 100 *μ*M) on 4xHFS-induced L-LTP in euploid control-derived slices and (b) Ts65Dn-derived slices. Magnification of the final 10 minutes of recording. Colored lines represent the mean fEPSP slopes calculated for the last 10 minutes of recording. Representative fEPSP traces showing severe oscillations recorded from control-derived slices and Ts65Dn-derived slices after being exposed to picrotoxin. (c) Summary graph of euploid littermate control L-LTP data and (d) Ts65Dn L-LTP data (mean fEPSP slope during the last 10 min for each recording condition). 10 and 100 *μ*M of picrotoxin rescued L-LTP to control levels in Ts65Dn-derived slices while causing severe oscillations in postsynaptic potentials. (e) Normalized data shows that 100 *μ*M picrotoxin had a greater effect on Ts65Dn-derived slices compared to control slices. *p* < 0.05 and *p* < 0.01 are represented by ^∗^ and ^∗∗^, respectively. Number of slices (animals) for control (without picro (*n* = 16), picro 0.1 *μ*m (*n* = 9), picro 1 *μ*m (*n* = 9), picro 10 *μ*m (*n* = 10), and picro 100 *μ*m (*n* = 9)) and Ts65Dn (without picro (*n* = 15), picro 0.1 *μ*m (*n* = 9), picro 1 *μ*m (*n* = 9), picro 10 *μ*m (*n* = 9), and picro 100 *μ*m (*n* = 9)). Error bars represent SEM. Arrows indicates LTP induction (4xHFS; with a 5 min intertrain intervals), representative traces show synaptic response during baseline (1) and at end of recording (2). Scale bars represent 2 mV (horizontal) and 10 ms (vertical).

Here, we tested the effects of 0.1, 1, 10, and 100 *μ*M picrotoxin on the levels of CA1 L-LTP in both Ts65Dn- and euploid control-derived hippocampal slices (Figures 4(a)–4(d)). At these concentrations, picrotoxin did not significantly affect the mean levels of L-LTP in euploid control-derived slices (*F*_(4,48)_ = 1.69, *p* = 0.17) (Figure 4(c)), but significantly increased the mean levels of L-LTP in Ts65Dn-derived slices (*F*_(4,45)_ = 3.45, *p* = 0.015) (Figure 4(d)). Post hoc analysis, however, revealed that picrotoxin only produced significant increases in the mean level of L-LTP in Ts65Dn-derived slices at the two highest concentrations, that is, 10 *μ*M (*p* = 0.0046) and 100 *μ*M (*p* = 0.00995) (Figure 4(d)).

In the same way that was done in the analysis of the previous experiment with memantine, data was normalized to the untreated genotype-specific levels (Figure 4(e)) as an alternative way to evaluate whether there was a genotype dependence for the picrotoxin effects. A RM-ANOVA showed significant genotype (*F*_(1,16)_ = 6.24, *p* = 0.024) and dose (*F*_(4,64)_ = 3.58, *p* = 0.011) effects, however, no significant interaction between dose and genotype was detected (*F*_(4,64)_ = 1.13, *p* = 0.35). Post hoc analysis showed that only 100 *μ*M picrotoxin produced a relatively greater response in Ts65Dn-derived slices when compared with euploid control slices (*p* = 0.040).

In Figures 4(a) (right panel) and 4(b) (right panel), we illustrate that the same concentrations of picrotoxin (10 and 100 *μ*M) that significantly enhance the levels of L-LTP in Ts65Dn mice also produce significant seizure-like oscillations in both pre- and posttetanic fEPSPs. Additionally, posttetanic fEPSPs displayed significant network oscillations at concentrations of picrotoxin as low as 1 *μ*M in Ts65Dn- and euploid control-derived slices.

### 3.4. Effects of A*β* and rPrP on 4xHFS-Induced LTP in Ts65Dn-Derived Hippocampal Slices

Soluble A*β* oligomers are potent synaptotoxins that have been shown to inhibit 4xHFS-induced LTP in the CA1 region of the hippocampus [31, 32]. Recently, Scott-McKean et al. [30] have shown that soluble rPrP and its N-terminal fragment N1 block this A*β* oligomer-induced inhibition of 4xHFS-induced E-LTP.

**Figure 5:**
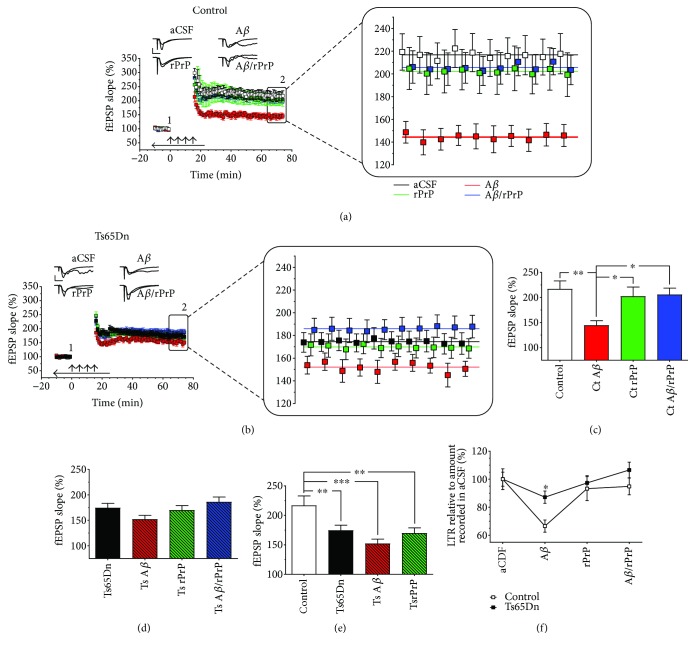
A*β* oligomers inhibit 4xHFS-induced LTP in euploid slices (but not Ts65Dn slices) and rPrP suppresses this inhibition. (a, b) Normalized fEPSP slopes in the presence or absence of A*β* oligomers alone or A*β* mixture with rPrP from euploid control-derived slices (a) or Ts65Dn-derived slices (b). Magnification of the final 10 minutes of recording. Colored lines represent the calculated mean fEPSP slopes for the last 10 minutes of recording. (c, d) Mean fEPSP slopes during the last 10 min of fEPSP recordings derived from data shown in panels (a) and (b), respectively. In Ts65Dn-derived slices, which already shows a deficit in 4xHFS-induced L-LTP when compared to euploid controls, A*β* oligomers had no further effect on L-LTP. rPrP rescued A*β* oligomer-induced inhibition 4xHFS-induced LTP in control slice; however, rPrP alone had no effect on 4xHFS-induced LTP in Ts65Dn-derived slices. (e) Shows that the levels of LTP in untreated control slices is significantly larger than the levels of LTP observed in Ts65Dn mouse slices under any treatment. (f) Relative decrease in 4xHFS-induced LTP due to A*β* oligomer-induced inhibition. Euploid control slices showed greater inhibition due to A*β* oligomers when compared to Ts65Dn-derived slices. *p* < 0.05, *p* < 0.01, and *p* < 0.001 is represented by ^∗^, ^∗∗^, ^∗∗∗^, respectively. Number of slices (animals) for control (aCSF (*n* = 14), A*β* (*n* = 10), rPrP (*n* = 11), and A*β* mixture with rPrP (*n* = 10)) and Ts65Dn (aCSF (*n* = 14), A*β* (*n* = 13), rPrP (*n* = 12), and A*β* mixture with rPrP (*n* = 12)). Error bars represent SEM. Arrows indicates LTP induction (4xHFS; with a 5 min intertrain intervals), representative traces show synaptic response during baseline (1) and at end of recording (2). Scale bars represent 1 mV (horizontal) and 10 ms (vertical). Horizontal black bar indicate aCSF, A*β*, rPrP, or A*β* mixture with rPrP.

In agreement with the work of Scott-McKean et al. [30], we found that superfusion of euploid control-derived hippocampal slices with A*β* oligomers (500 nM) produced a noticeable inhibition of 4xHFS-induced LTP (Figures 5(a) and 5(c)) in relation to untreated slices from these same animals. Furthermore, when slices were exposed to A*β* (500 nM) mixed with rPrP (100 nM), this inhibition was suppressed (Figure 5(c)), which is reflected by the statistically significant treatment effect observed (*F*_(3,41)_ = 4.31, *p* = 0.0099). Post hoc analysis indicated that A*β* oligomers significantly decreased the level of 4xHFS-induced LTP in euploid control-derived slices (*p* = 0.0015) (Figure 5(c)) and that soluble rPrP restored the mean level of LTP to a value statistically indistinguishable from that of untreated slices (*p* = 0.60). Unlike in control-derived hippocampal slices, however, there was little to no effect of A*β* oligomers on the induction of 4xHFS-induced LTP in Ts65Dn-derived slices (Figures 5(b) and 5(d)). Indeed, a one-way ANOVA found no significant treatment effect with A*β*, soluble rPrP, or A*β* plus rPrP on the mean levels of 4xHFS-induced LTP in Ts65Dn-derived slices (*F*_(3,47)_ = 2.51, *p* = 0.070).

We then compared directly the mean level of LTP in control slices with those of untreated and treated Ts65Dn-derived slices. To this end, we performed the comparison illustrated in Figure 5(e) and an additional ANOVA, which produced a quantitative measure of statistical significance of the observed differences in 4xHFS-induced LTP (*F*_(3,49)_ = 6.17, *p* = 0.0012). Post hoc analysis showed significantly decreased levels of 4xHFS-induced LTP in untreated Ts65Dn-derived slices (*p* = 0.0084), A*β* oligomer-treated Ts65Dn-derived slices (*p* = 0.00014), and rPrP-treated Ts65Dn-derived slices (*p* = 0.0051) when compared to euploid hippocampal slices.

Finally, data was normalized once again to the untreated genotype-specific levels (Figure 5(f)). A two-way ANOVA detected significant genotype (*F*_(1,87)_ = 4.87, *p* = 0.030) and treatment (*F*_(3,87)_ = 6.79, *p* = 0.00036) effects, but no significant interaction between treatment and genotype (*F*_(3,87)_ = 1.20, *p* = 0.32). Post hoc analysis confirmed that control-derived slices displayed a greater inhibition to 4xHFS-induced LTP by A*β* oligomers when compared with Ts65Dn-derived slices (*p* = 0.00029).

## 4. Discussion

In this study, we examined three representative modalities of LTP in the CA1 region of hippocampal slices obtained from the Ts65Dn mouse model of DS. The first goal of this study was to test the hypothesis that a therapeutically relevant concentration of memantine (1 *μ*M) [33] might interfere negatively with the induction and/or maintenance of LTP, and, by extension, with fundamental cellular mechanisms underlying learning and memory in these animals. We had to consider this hypothesis because of all accumulated evidence that the proper function of NMDA receptors is critical for the induction of LTP [34–36]. There was also previous work demonstrating that the same concentration of memantine rescues the exaggerated levels of NMDA receptor-dependent CA1 LTD in Ts65Dn mice by reducing the mean levels of this form of synaptic plasticity [17]. In light of preclinical and clinical work underway aimed at testing this NMDA receptor antagonist as a potential pharmacological intervention to enhance the cognitive abilities of individuals with DS, this issue has assumed particular importance, given that an inhibitory action of memantine on LTP could potentially translate into undesirable clinical effects of this drug.

Here, it is important to notice that, although memantine's mechanism of action is generally assumed to primarily due to its antagonism of NMDA receptor channels, it should be noted that, because memantine also binds to a number of other receptors, a few alternative mechanisms for its action have been proposed. For example, Moriguchi et al. [37] proposed that ATP-sensitive K^+^ (K_ATP_) channels are potentially relevant targets for memantine action in the treatment of AD.

For the two forms of CA1 LTP that had already been investigated in Ts65Dn mice, our results from untreated slices were consistent with previous reports [14, 38–40]. We found no significant difference in the mean levels of HFS-induced LTP between Ts65Dn- and euploid control-derived slices, whereas a small but significant reduction in the mean level of TBS-induced LTP was observed in Ts65Dn mouse-derived slices compared with that of euploid control animals. Contrary to our working hypothesis, however, we found that memantine not only had no inhibitory effect on LTP induced by HFS, but it actually rescued the deficit in TBS-induced LTP in Ts65Dn mice. Indeed, this pharmacological rescue of TBS-induced LTP may be, in addition to the rescue of exaggerated levels of NMDA-dependent LTD, another electrophysiological correlate of memantine's memory and learning enhancing effects in Ts65Dn mice.

In addition to the HFS- and TBS-induced modalities of LTP, we also assessed CA1 LTP induced by 4xHFS, which is a form of stimulation capable of producing the highly durable form of synaptic plasticity known as L-LTP. In addition to requiring gene transcription and protein synthesis in the postsynaptic neuron, this form of LTP is regulated by the activity of the regulator of calcineurin 1, RCAN1 (also known as Down syndrome critical region 1 (DSCR1)) [41], which is a gene product of chromosome 21 whose orthologue is also located in the Ts65Dn chromosome. This observation was critical in motivating the assessment of L-LTP in Ts65Dn mice here, given that dysfunction of NMDA receptors in DS was originally theoretically predicted as the potential consequence of the inhibition of calcineurin activity due to overexpression of chromosome 21 gene products such as RCAN1 and DYRK1A (perhaps coupled to elevated amounts of reactive oxidative species) [42]. Therefore, the finding of reduced levels of CA1 LTP induced by 4xHFS recorded at both early phase and late phase in hippocampal slices from Ts65Dn when compared with similar recordings made from euploid littermate control-derived slices was both an almost predictable finding and a key piece of the puzzle toward a better understanding of DS pathophysiology at the synaptic level.

The uncomplicated argument that one can make for the observed genotype dependence of LTP induced by 4xHFS would be that calcineurin activity might have an inverse U-shaped effect on L-LTP. This would mean that in both Ts65Dn and RCAN1 knockout mice, dysregulated levels of calcineurin activity might produce suboptimal levels of L-LTP. However, if such effect was the result of alterations in NMDA receptor function, the expectation would be that memantine might also pharmacologically rescue these lower-than-control levels of synaptic potentiation. Instead, we found that memantine, even when superfused at a level 10 times higher than the therapeutically relevant concentration of 1 *μ*M, had no significant effect on the mean level 4xHFS-induced LTP in Ts65Dn mouse-derived slices. We also found that, although memantine at a concentration of 1 *μ*M had no effect on L-LTP in control-derived slices, at 3 and 10 *μ*M, it started to inhibit this form of LTP in slices from euploid mice. In its own way, this differential, genotype-dependent effect of memantine on L-LTP provides additional support to the hypothesis that NMDA receptor function is altered in this mouse model of DS. However, instead of being more susceptible to undesirable effects of potentially toxic doses of memantine, as far as inhibition of L-LTP goes, Ts65Dn mice have shown to be more resistant to high doses of memantine than euploid control mice.

Given that this was the first exploration of the properties of 4xHFS-induced LTP in Ts65Dn mice, the second goal of this study was to quantify the effects of a few additional molecules of interest to the study of DS that could potentially shed some light on this specific form of LTP in Ts65Dn mice. These molecules were (1) picrotoxin; (2) A*β* oligomers; and (3) soluble rPrP.

The idea that the inhibition of GABA_A_ receptors could enhance LTP in brain slices from Ts65Dn mice was first explored by Kleschevnikov et al. [13], who showed that 100 *μ*M picrotoxin can increase the mean levels of LTP in the dentate gyrus to the same level as those in slices from euploid control mice. A similar effect was then described by Costa and Grybko [14], who demonstrated that 10 *μ*M picrotoxin could also elevate the mean level of TBS-induced CA1 LTP in Ts65Dn-derived slices to those seen in euploid control-derived slices. Subsequently, work by Fernandez et al. [43] showed that Ts65Dn mice treated with a chronic, 2-week daily regimen of the subconvulsive dose of 1 mg/kg picrotoxin intraperitoneally (i.p.) displayed a durable enhancement in object recognition performance. This i.p. dose of picrotoxin (which is a 1 : 1 molecular complex of picrotin and picrotoxinin) in the rat (data is not available in the mouse) translates into peak serum levels of ≈2 *μ*M of picrotin and picrotoxinin, which are reached in ≈30 min and rapidly decay to baseline levels in ≈60 min [44].

In the present study, we assessed the effects of 0.1, 1, 10, and 100 *μ*M of picrotoxin on 4xHFS-induced CA1 L-LTP in Ts65Dn-derived hippocampal slices. We found that picrotoxin produced significantly increases in the mean level of L-LTP in Ts65Dn-derived slices at 10 and 100 *μ*M, but not at lower concentrations. However, these increases in the mean levels of LTP were also accompanied by high-amplitude 200–250 Hz network oscillations in slices of both genotypes, which could be detected before and after LTP induction (see analysis of oscillations in Supplementary Data available here). Therefore, given these seizure-like oscillations produced by high doses of picrotoxin, the term “pharmacological rescue” of the LTP levels can hardly be applied to this treatment. Indeed, 10 *μ*M picrotoxin is similar to the peak serum levels of this compound that are achieved after typical seizure-inducing doses of 3–5 mg/kg i.p. [44, 45]. To date, the positive effects on memory and learning performance found after a chronic regimen of the subconvulsive doses of picrotoxin in Ts65Dn mice remain unexplained, but are unlikely to be due to acute enhancements in the levels of any form of CA1 LTP. This same line of reasoning is also likely to apply to other modulators of GABA_A_ receptors, such as pentylenetetrazole or RG1662.

This pharmacological survey of the properties of 4xHFS-induced CA1 E-LTP in Ts65Dn-derived hippocampal slices concluded with the investigation of the effects of A*β* oligomers and soluble rPrP. The obvious reason to probe the effects of A*β* oligomers on LTP induced by 4xHFS in Ts65Dn-derived slices was that the amyloid precursor protein gene, *APP* (which encodes the protein from which A*β* peptides originate), is present in three copies in persons with DS and Ts65Dn mice. Indeed, trisomy of *APP* is thought to be a necessary molecular factor underlying the virtually universal incidence of AD-type pathology in persons with DS aged 40 year and beyond [46]. Additionally, different studies have demonstrated that A*β* oligomers can inhibit 4xHFS-induced LTP in the CA1 region of the hippocampus [30–32]. These findings made it particularly attractive to investigate the effects of A*β* oligomers in Ts65Dn mice, which are likely to be constitutively exposed to higher-than-typical levels of these oligomers.

We had two competing hypotheses in designing the experiments involving the effects of A*β* oligomers and soluble rPrP on 4xHFS-induced CA1 E-LTP. First hypothesis: 4xHFS-induced CA1 LTP in hippocampal slices from Ts65Dn mice would be more sensitive to A*β* oligomers than in slices from euploid control mice due to the additive effects of exogenous and endogenous A*β* oligomers. Second hypothesis: 4xHFS-induced CA1 LTP would be less sensitive in slices from Ts65Dn mice versus those from euploid control animals, due to compensatory developmental mechanisms. In the same vein of thought, the use of soluble rPrP allowed us to interrogate directly whether the reduced levels of 4xHFS-induced CA1 LTP observed in Ts65Dn-derived slices were directly due to effect of elevated endogenous A*β* oligomers in the brains of these animals, given that this molecule is known to block the A*β* oligomer-induced inhibition of LTP [30].

Our results showing that A*β* oligomers cause substantially stronger inhibition of 4xHFS-induced LTP in control-derived slices when compared with Ts65Dn-derived slices are consistent with the second hypothesis, suggesting that hippocampi in Ts65Dn mice might have developed resistance to exogenous A*β* oligomers, likely due to compensatory developmental mechanisms. Whether these mechanisms are in any way related to a potential long-term exposure to elevated levels of endogenous A*β* remains to be determined. Nevertheless, the present data clearly indicate that the intrinsically depressed level of LTP in Ts65Dn-derived slices cannot be rescued by rPrP, a protein that blocks the exogenous A*β* oligomer-mediated inhibition of LTP in slices from euploid control animals.

As pointed out in the introduction of this research article, the quantification of 4xHFS-induced LTP can be seen as an informative assay of the integrity of the essential transcriptional and translational machinery in neurons from different mouse models of neurological and psychiatric disorders. Therefore, the findings regarding this form of LTP in Ts65Dn that we presented here have important consequences from both basic and translational points of view. From the basic science aspect, it describes an animal model of a human disorder in which the integrity of transcription and/or translation is compromised and provides a new useful tool for future in depth research in these areas in mouse and human cell-based models of DS. From the translational perspective, it is somewhat refreshing to see a phenotype in Ts65Dn that is resistant to pharmacological intervention. Recent reviews of preclinical studies in Ts65Dn mice have described more than a dozen pharmacological or nutritional interventions that have rescued electrophysiological and/or behavioral phenotypes [9]. However, even for compounds or drugs for which we have had solid, reproducible, and powerful effects in the mouse, such as memantine, small scale clinical research in human beings, although promising, have shown at best mild-to-moderate effect sizes in very specific domains of cognition. Therefore, revisiting some of the historically successful preclinical agents used in Ts65Dn mice with 4xHFS-induced LTP (as we have done here for memantine, picrotoxin, and rPrP) may reveal a more nuanced picture in which many of those agents may have no effect, some may have negative effects and some may rescue this synaptic plasticity phenotype without causing seizure-like network oscillations.

In recent years, there has been a growing availability of new mouse models of DS, some of which were designed to address the shortcomings of the Ts65Dn mouse model (for reviews see [9, 10]). These shortcomings include: (1) incompleteness of the set of chromosome 21 orthologous genes triplicated in the Ts65Dn marker chromosome and (2) unspecificities in the set of genes contained in the Ts65Dn trisomic segment. A frequently cited unspecificity is that Ts65Dn mice are also trisomic for a >5.8 Mb subcentromeric region on mouse chromosome 17 (which contains at least 50 genes) that is not orthologous to any region on human chromosome 21 [47]. Another unspecificity comes from the fact that the Ts65Dn trisomic segment includes many mouse-specific genes not represented in the human genome. In this context, the present study provides a necessary baseline for a new LTP phenotype that can be explored in future studies involving neural plasticity in these newer and emerging mouse models of DS, including overexpressing single gene models such as *Girk2*, *App*, *Dyrk1a*, *Rcan1*, and *Olig2* transgenic mice. In addition, correction of gene copy number for some of these genes, which are present in three copies in Ts65Dn mice, has been shown to result in the correction of some forms of synaptic plasticity alteration in these animals.

## 5. Conclusions

In this study, we learned that both E-LTP and L-LTP induced by 4xHFS were significantly depressed when compared with these same forms of LTP in euploid control slices. In addition, the drug memantine, when used at the pharmacologically relevant concentration of 1 *μ*M, had no detectable effect on HFS LTP or 4xHFS-induced L-LTP in the mouse model of DS Ts65Dn or control-derived slices, but this same concentration of memantine rescued TBS LTP in Ts65Dn-derived slices to control euploid levels. Therefore, at therapeutic levels, memantine produces no adverse effects on the induction or maintenance of LTP in hippocampal slices from Ts65Dn mice and actually enhances the levels of one modality of LTP in slices from this mouse model of DS. We also investigated the effects of picrotoxin, amyloid beta oligomers, and soluble (membrane anchor-free) recombinant human rPrP on this specific form of LTP. Whereas 10 *μ*M and 100 *μ*M of picrotoxin increased the mean levels of L-LTP in Ts65Dn-derived slices to values comparable to those in euploid control slices, these concentrations of picrotoxin also produced seizure-like oscillations in postsynaptic potentials. Hence, this (and previously reported) pharmacologically induced enhancements on the levels of LTP in Ts65Dn mice cannot be called a true pharmacological rescue, given that any potential benefit of such high levels of GABA_A_ receptor inhibition would be offset by the induction of seizures. Finally, amyloid beta oligomers did not significantly inhibit the levels of 4xHFS-induced E-LTP in Ts65Dn-derived slices, and rPrP alone had no effect on E-LTP on either Ts65Dn-derived or euploid control-derived slices.

## Data Availability

All data arising from this study are contained within the manuscript.

## Abbreviations

DS: Down syndrome
LTP: Long-term potentiation
L-LTP: Late-phase LTP
LTD: Long-term depression
HFS: High-frequency stimulation
TBS: Theta-burst stimulation
rPrP: Recombinant human prion protein
NMDA: N-Methyl-D-aspartic acid
GABA: Gamma-aminobutyric acid.

## Supplementary Materials

Supplementary Materials Input/output (I/O) function, paired-pulse facilitation (PPF), and quantitative analysis of picrotoxin-induced oscillations.
