# Supplementary material for: Pharmacological Modulation of Three Modalities of CA1 Hippocampal Long-Term Potentiation in the Ts65Dn Mouse Model of Down Syndrome
Source: Neural Plast. 2018 Apr 10;2018:9235796. doi: 10.1155/2018/9235796 (PMC5914153; doi:10.1155/2018/9235796)
Supplement: Supplementary Materials — Input/output (I/O) function, paired-pulse facilitation (PPF), and quantitative analysis of picrotoxin-induced oscillations. [file 9235796.f1.doc]

**Supplementary Materials**

**Input/Output (I/O) Function and Paired-pulse Facilitation (PPF)**

**Results**

In order to investigate potential effects of memantine on basic synaptic function of Ts65Dn and euploid control mice, we performed assessments of input/output (I/O) function and paired-pulse facilitation (PPF) for the eight groups of hippocampal slices, i.e., untreated control, control preincubated with 1, 3, and 10 μM of memantine, untreated Ts65Dn, and Ts65Dn preincubated with 1, 3, and 10 μM of memantine. Analysis of the effect of memantine on I/O function by repeated measures ANOVA for euploid control mice (Figure S1 (a)) showed no significant drug differences (F_(3,79)_ = 0.7177; P = 0.5443). and no significant interaction between memantine dose and stimulus intensity (F_(27,711)_ = 1.2844; P = 0.1533). As expected, there was a significant stimulus intensity effect (F_(9,711)_ = 257.0067; P < 0.001). For Ts65Dn mice (Figure S1 (b)), analysis of the I/O function plot by repeated measures ANOVA showed no significant drug differences (F_(3,80)_ = 0.1664; P = 0.9187), and no significant interaction between memantine dose and stimulus intensity (F_(27,720)_ = 1.1825; P = 0.2404). However, there was a significant stimulus intensity effect (F_(9,720)_ = 210.6426; P < 0.001).

We also investigated of the effect of memantine on PPF, which is a form of short-lived plasticity sensitive to presynaptic changes. In a subset of slices, PPF was produced by delivering two stimuli of identical strength at different interpulse intervals to assess the integrity of presynaptic mechanisms in Ts65Dn mice. PPF was calculated as the slope ratio between the second and first stimuli. Analysis of the data for euploid control mice (Figure S1 (c)) showed no significant drug effect (F_(3,48)_ = 0.294; P = 0.8295) or interaction between memantine and interpulse interval (F_(21,336)_ = 0.626; P = 0.8994) in the range tested. A strongly significant interpulse interval effect was found (F_(7,336)_ = 88.031; P < 0.001). Ts65Dn mice (Figure S1 (d)) also showed no significant drug effect (F_(3,37)_ = 1.214; P = 0.3182) or interaction between memantine and interpulse interval (F_(21,259)_ = 1.056; P = 0.3968). As with control mice, the Ts65Dn mice showed a significant interpulse interval effect was found (F(7,259) = 216754; P < 0.001).

We next evaluated I/O function and PPF for aCSF, Aβ, rPrP, or Aβ/rPrP on hippocampal slices from Ts65Dn and euploid littermate control mice. Analysis of the I/O function by repeated measures ANOVA for euploid control mice (Figure S2 (a)) showed no significant differences between the test molecules (aCSF, Aβ, rPrP, or Aβ/rPrP) (F_(3,44)_ = 2.4124; P = 0.0794). However, it did indicate a significant interaction between test molecules and stimulus intensity (F_(27,396)_ = 2.165; P = 0.000834), and a significant stimulus intensity effect (F_(9,396)_ = 182.1021; P < 0.001). For Ts65Dn mice (Figure S2 (b)) analysis of the I/O function plot by repeated measures ANOVA showed no significant differences the test molecules (aCSF, Aβ, rPrP, or Aβ/rPrP) (F_(3,46)_ = 0.3654; P = 0.7783), and no significant interaction between the test molecules and stimulus intensity (F_(27,414)_ = 0.2911; P = 0.9999). However, there was a significant stimulus intensity effect (F_(9,414)_ = 149.7814; P < 0.001). Fisher’s LSD post-hoc test showed a significant difference between control slices perfused with only aCSF and control slices perfused with rPrP at stimulus intensity 100 μA (p=0.04366) (Figure S2 (a)), and a significant difference between control slices perfused with only aCSF and control slices perfused with Aβ/rPrP at stimulus intensities 60, 70, 80, 90, and 100 μA (p=0.02186, p=0.004402, p=0.000881, p=0.000121, and p=0.000024, respectively) (Figure S2 (a)). Fisher’s LSD post-hoc test also showed a significant difference between control slices perfused with Aβ oligomers and control slices perfused with Aβ/rPrP at stimulus intensities 70, 80, 90, and 100 μA (p=0.04461, p=0.01997, p=0.007644, and p=0.00413, respectively) (Figure S2 (a)), and showed a significant difference between control slices perfused with rPrP and control slices perfused with Aβ/rPrP at stimulus intensities 90, and 100 μA (p=0.04507, and p=0.02821, respectively) (Figure S2 (a)).

Analysis of these test molecules (aCSF, Aβ, rPrP, or Aβ/rPrP) on PPF for euploid control mice (Figure S2 (c)) showed no significant effect of test molecules (F_(3,50)_ = 0.430; P = 0.7326) or interaction between the test molecules and interpulse interval (F_(21,350)_ = 0.509; P = 0.9664). A strongly significant interpulse interval effect was found (F_(7,350)_ = 34.122; P < 0.001). Ts65Dn mice (Figure S2 (d)) also showed no significant effect test molecules (aCSF, Aβ, rPrP, or Aβ/rPrP) (F_(3,38)_ = 1.423; P = 0.2510) or interaction between test molecules and interpulse interval (F_(21,266)_ = 0.988; P = 0.4789). Ts65Dn mice showed a significant interpulse interval effect was found (F_(7,266)_ = 87.046; P < 0.001).

**Figure S1**

**
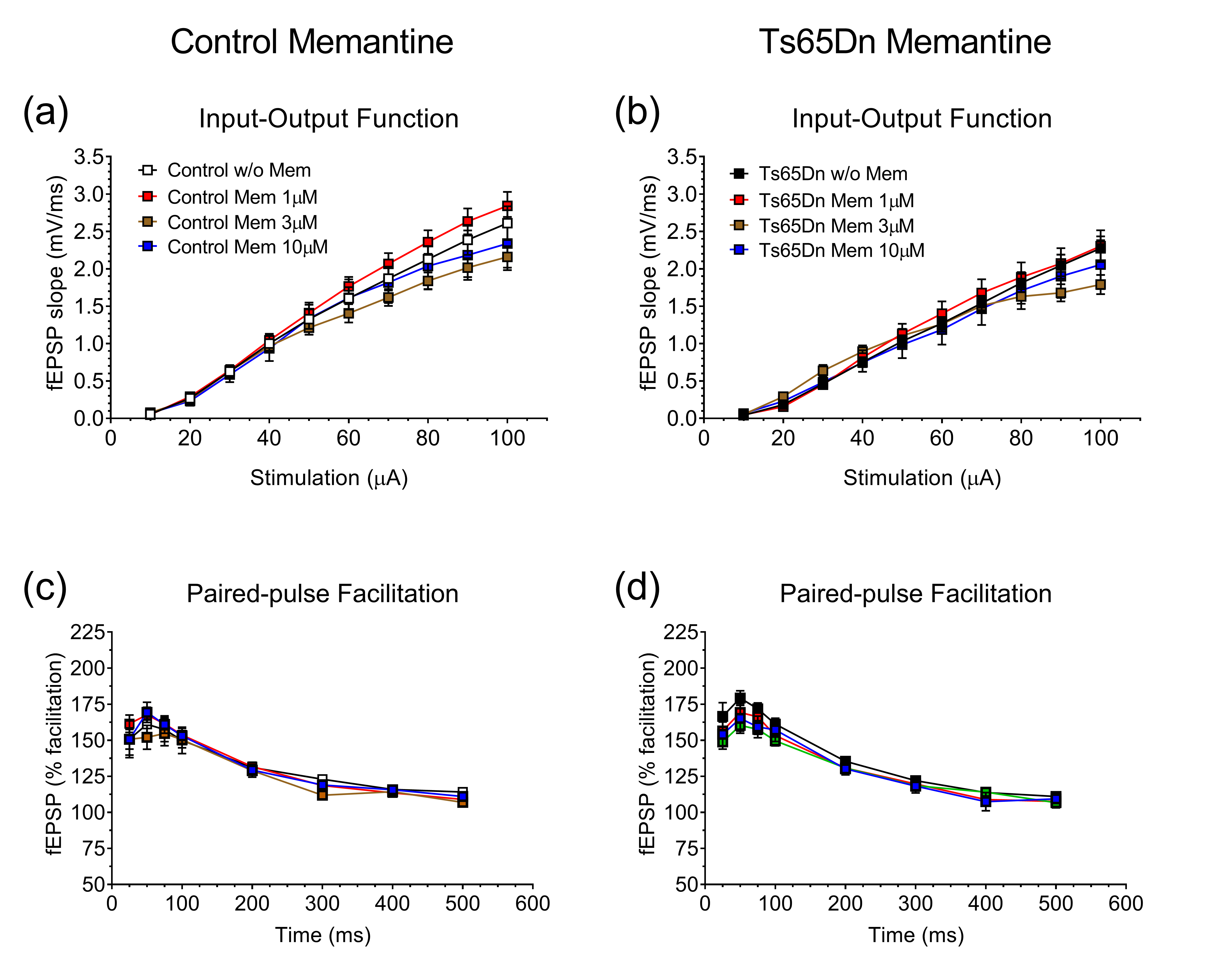
**

**Figure S2**

**
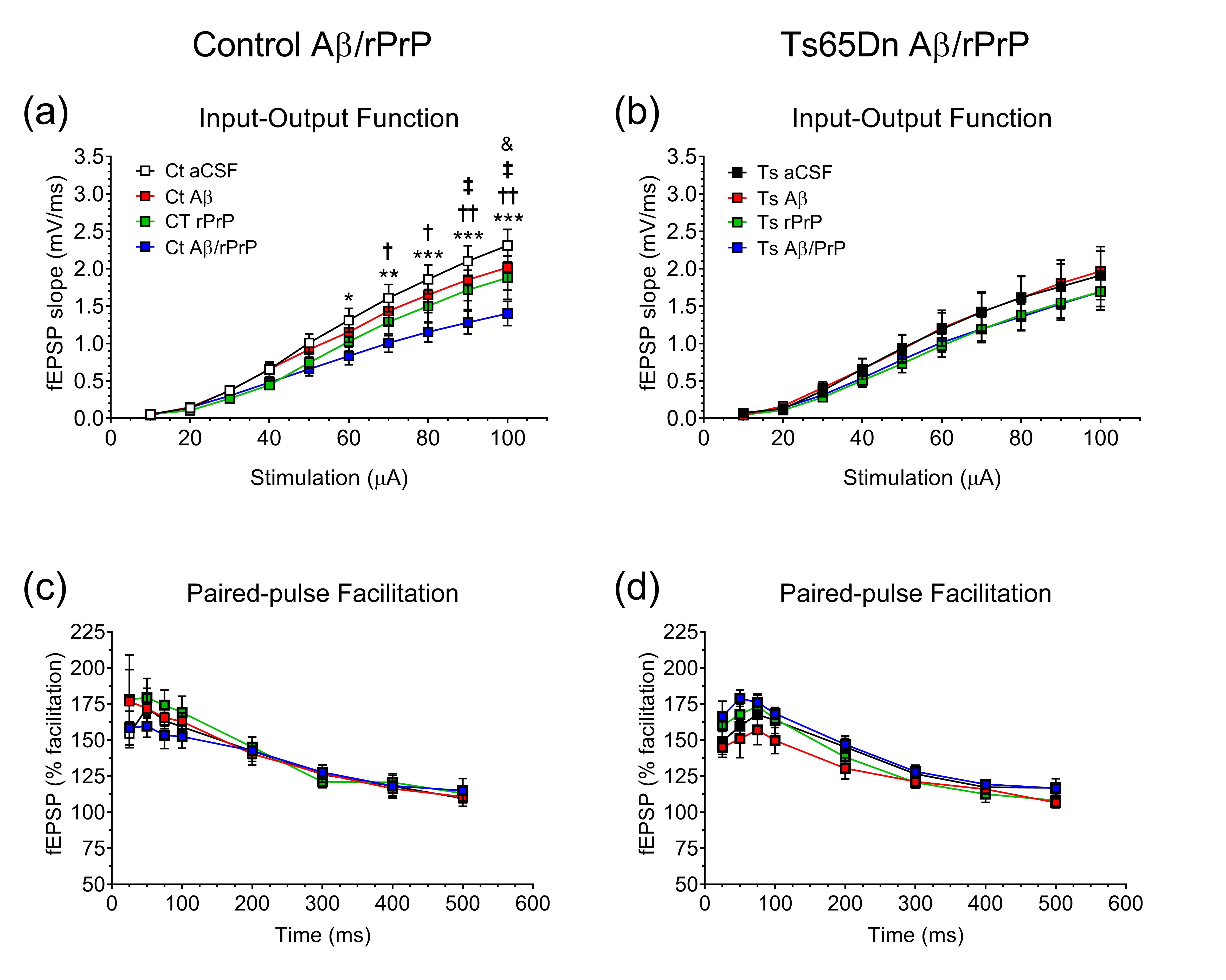
**

**Picrotoxin-induced Oscillations**

**Methods**

Each recording sample from experiments in which we investigated the actions of picrotoxin was analyzed for oscillatory characteristics. For each sample, the 10 sweeps were ensemble averaged for baseline (10 sweeps just prior to stimulation) and 170-180 minutes post-LTP induction (10 sweeps). Because the oscillations were superimposed on the stimulus response, a time-domain based assessment of the oscillations was implemented. The method sequentially traced positive and negative peaks in the signal and its time derivative creating four time points or quadrants per oscillatory cycle. Since there may be multiple local peaks, a second pass was used to find the global peak within each quadrant. The cycles included the initial stimulus artifact, the primary evoked response, and then possibly one or more oscillatory cycles. Each oscillatory cycle was visually validated, and up to two cycles per trace were selected for the analysis. Traces with no apparent oscillations were excluded from the analysis. Four time points for each oscillatory cycle were used to calculate the period (peak-to-peak, valley-to-valley, rising edge-to-rising edge, and falling edge-to-falling edge). For each trace, the period was taken as the median of all the available periods. The amplitude was calculated at each selected peak-to-valley and valley-to peak, and the median was used as the amplitude for the trace.

**Results**

In comparisons of recordings from Ts65Dn with euploid control mice at each concentration of picrotoxin, the only significant difference was at 10 µM, where control sample oscillations were lower amplitude (p=0.0036) and slightly slower (p=0.034) (See Table I). These are the conditions where samples were near the threshold for oscillation, and some samples showed lower than typical amplitude oscillations.

Table II shows the overall summary of oscillations, combining both Ts65Dn and control euploid groups and all traces at all picrotoxin concentrations that exhibited oscillations. There was no significant difference between baseline and post-LTP induction traces in mean period or equivalent frequency. There was, however, a significantly larger amplitude in the post-LTP induction traces compared to baseline (p=0.00026). Overall, the traces that showed oscillation demonstrated a remarkable consistency in the oscillation frequency.

Table III shows the percentage of traces at each picrotoxin concentration and at baseline and post-LTP induction conditions. At 0.1-µM picrotoxin, few samples oscillated, and only post-LTP induction. At 1-µM picrotoxin, no samples oscillated at baseline but most did post-LTP induction. At 10 and 100 µM, most samples oscillated at baseline, and all samples oscillated post-LTP induction.

| **Table I. Comparison of Oscillations between Ts65Dn and Control groups (10 µM picrotoxin at baseline)** | | | |
| --- | --- | --- | --- |
|  | **N** | **Amplitude (mV)**  **Mean±SE** | **Period (msec)**  **Mean±SE** |
| **Control** | 10 | 0.472±0.077 | 4.83±0.229 |
| **Ts65Dn** | 7 | 0.930±0.114 | 4.18±0.160 |
| Amplitude difference was significant at p=0.0036  Period difference was significant at p=0.034 | | | |

| **Table II. Picrotoxin Oscillations** | | |
| --- | --- | --- |
| **Period (msec)** | **N** | **Mean±SE** |
| at Baseline | 34 | 4.47±0.080 |
| at LTP | 57 | 4.32±0.066 |
| Overall | 91 | 4.38±0.051 |
|  |  |  |
| **Equivalent Frequency (Hz)** | **N** | **Mean±SE** |
| At baseline | 34 | 223.7±4.1 |
| Post-LTP induction | 57 | 231.5±3.6 |
| Overall | 91 | 228.3±2.7 |
|  |  |  |
| **Amplitude (mV)** | **N** | **Mean±SE** |
| At baseline | 34 | 0.672±0.052 |
| Post-LTP induction | 57 | 0.927±0.042 |
| Amplitude difference was significant at p=0.00026 | | |

| **Table III. Percentage of samples exhibiting oscillation** | | |
| --- | --- | --- |
| **Picrotoxin**  **Concentration (µM)** | **at Baseline** | **at LTP** |
| 0.1 | 0.0% (0 of 18) | 16.7% (3 of 18) |
| 1 | 0.0% (0 of 18) | 88.9% (16 of 18) |
| 10 | 80.0% (16 of 20) | 100.0% (20 of 20) |
| 100 | 94.4% (17 of 18) | 100.0% (18 of 18) |
| Overall | 61.5% (91 of 148) | |
